# Microbubble drug conjugate and focused ultrasound blood brain barrier delivery of AAV-2 SIRT-3

**DOI:** 10.1080/10717544.2022.2035855

**Published:** 2022-04-08

**Authors:** Dennison Trinh, Joanne Nash, David Goertz, Kullervo Hynynen, Sharsi Bulner, Umar Iqbal, James Keenan

**Affiliations:** a Department of Biological Sciences, University of Toronto at Scarborough, Scarborough, Canada; b Sunnybrook Research Institute, Toronto, Canada; c Department of Medical Biophysics, University of Toronto, Toronto, Canada; d Human Health Therapeutics Research Centre, National Research Council of Canada, Ottawa, Canada; e Artenga Inc, Ottawa, Canada

**Keywords:** Focused ultrasound, microbubbles, BBB drug delivery, Sirtuin3, SIRT3, Parkinson’s

## Abstract

**Background:**

Delivery of viral vectors as gene therapies to treat neurodegenerative diseases has been hampered by the inability to penetrate the blood brain barrier (BBB) and invasive or non-targeted delivery options prone to inducing immune responses. MR guided focused ultrasound (MR-g-FUS) and microbubbles have demonstrated safe, temporary, targeted BBB permeabilization clinically.

**Methods:**

We developed clinically scalable, microbubble drug conjugates (MDCs) for the viral gene therapy, AAV.SIRT3-myc [adeno-associated virus expressing myc-tagged SIRT3], which has previously been shown to have disease modifying effects in animal models of Parkinson’s disease (PD). The lipid shells of the perfluorocarbon gas MDCs were covalently conjugated to antibodies with binding specificity to AAVs. Following systemic (iv) delivery of AAV.SIRT3-myc MDCs, MR-g-FUS was used to deliver SIRT3-myc to brain regions affected in PD. SIRT3-myc expression was determined post mortem, using immunohistochemistry.

**Results:**

An *in vitro*, SH-SY5Y cell culture model was used to show that the localized destruction of MDCs using ultrasound exposures within biological safety limits dissociated AAV2-GFP (green fluorescent protein) from the MDCs in the targeted area while maintaining their transduction capacity. In rats, MR-g-FUS resulted in BBB permeabilization in the striatum and substantia nigra (SNc). SIRT3-myc was expressed in the striatum, but not the SNc.

**Conclusion:**

These studies demonstrate that MDCs combined with MR-g-FUS are an effective method for delivery of viral vector gene therapies, such as AAV.SIRT3, to brain regions affected in PD. This technology may prove useful as a disease-modifying strategy in PD and other neurodegenerative disorders.

## 1. Introduction

For neurological disorders, systemic administration of gene therapies is not effective, as the blood–brain barrier (BBB) prevents therapeutic levels reaching the brain, therefore, direct brain infusion is required. Direct brain infusion is especially invasive in the elderly, making it a less safe option. Furthermore, pathology for diseases such as Parkinson’s disease (PD) affect multiple brain regions, therefore disease-modifying therapy should target more than one brain region.

Magnetic resonance–guided-focused ultrasound (MR-g-FUS) combined with microbubbles circulating in the bloodstream provides a noninvasive method to enable safe delivery of gene therapy to specific brain regions affected in PD. The focused ultrasound is capable of targeting deep seated brain tissue to open up precise regions of the blood brain barrier in a safe, reversible and repeatable manner in both rats and primates (McDannold et al., 2012) and for glioblastoma, Alzheimer’s Disease, ALS, PD, and breast cancer brain met patients (Mainprize et al., 2019).

Experimental evidence suggests that the interaction between the ultrasound, microbubbles and brain vasculature leads to mechanical forces that temporarily open up tight junctions and induces active transport processes (Burgess et al., 2015) allowing increased uptake of systemic drugs to the brain parenchyma.

Alternate methods are available for the delivery of gene therapies to multiple regions of the brain, such as intra-cisterna magna and intravenous delivery, but, unlike MR-g-FUS mediated BBB permeabilization, these routes do not allow delivery to specific brain regions affected.

AAV9 gene therapy penetrates the BBB at a higher efficiency than other AAV serotypes (Lin et al., 2020) and has progressed to clinical trials to treat CNS disorders. However, AAV9 does not locally target diseased brain regions, does not transduce neurons as efficiently or at as low a dose as AAV2 and focused ultrasound (Stavarache et al., 2018), and can induce an immune response requiring an immuno-suppression regime (NCT04127578). These factors may reduce the patient population treatable by AAV9 gene therapy.

Viral gene therapy delivery to the brain has been demonstrated preclinically using MRgFUS and sequential intravenous (IV) administration of microbubbles and AAV gene therapy (Hynynen et al., 2019).

Conjugating drugs onto microbubbles has advantages over the sequential administration of drugs and microbubbles. A higher dose of the drug is delivered to the area of interest (Qian et al. (2019) and Wang et al. (2012)), there is enhanced drug perfusion and retention in diseased regions (Zhang et al., 2018), and systemic side effects may be mitigated as unused drug is cleared along with the microbubble lipid shells in the liver (Yanagisawa et al., 2007).

As well, in the free form, gene-based molecules have a very short half-life in physiological conditions owing to their vulnerability for degradation by endogenous nucleases. Microbubble conjugation of adenoviruses, which induces a higher immune response than AAV, has been shown to prevent innate and adaptive immune responses (De Carlo et al., 2019) *in vivo*. Adenovirus induces a transient gene expression compared to AAV. As well, the De Carlo study used biotin avidin conjugation technology not suitable for humans. Therefore conjugation of AAV gene therapy to lipid shelled microbubbles using clinically scalable conjugation methods has the potential to induce long lasting gene expression and to address rapid renal clearance, poor cellular uptake, degradation by endogenous enzymes, and immune response after systemic administration.

In addition, preexisting immunity against specific adeno-associated virus (AAV) serotypes or other viral gene therapies can result in the exclusion of patients from clinical trials. For example, an average prevalence for anti-AAV8 (∼40%) and anti-AAV5 (∼30%) neutralizing antibodies (Kruzik et al., 2019).

Thus there exists a need to develop a gene therapy delivery system for a variety of AAV serotypes to provide options for patients with preexisting immunity to specific serotypes. The method to develop AAV2-SIRT3 microbubble drug conjugates (MDCs) for MR-g-FUS mediated delivery described in this study may be used to develop MDCs for a variety of viral vectors, for example, AAV2, AAV9, lentiviral, HSV, which could potentially be used to treat a variety of neurological disorders, including neurodegenerative diseases.

PD is the second most common neurodegenerative disease and affected 6.1 million globally in 2016 vs. 2.5 million in 1990 (GDB 2016 Parkinson’s Disease Collaborators, 2018, *The Lancet Neurology*). Currently, only symptomatic treatments are available, and these are associated with side-effects, as well as having no effect on disease progression. We have shown previously that the gene therapy, AAV.SIRT3-myc [recombinant adeno-associated virus serotype over-expressing myc-tagged Sirtuin 3] is both neurorestorative and neuroprotective in rat models of Parkinson’s disease (PD; Gleave et al., 2017; Gleave et al., 2018). However, previous studies involved direct infusion of AAV.SIRT3-myc to the brain, which is a suboptimal delivery method clinically.

## 2. Methods

### 2.1. Lipid microbubble vesicles preparation

Lipid microbubble vesicles were prepared by dissolving DSPE-PEG (2000)-amine (1,2-Distearoyl-*sn*-Glycero-3-Phosphoethanolamine-N [amino(Polyethylene Glycol) 2000) and DSPC (1,2-Distearoyl-sn-glycero-3-phosphocholine) lipids (ratio 1.6:1 by mass) and chloroform, and sonicating at 60 °C in sonicating water bath. The solution was evaporated to produce a thin lipid film that was then solubilized in propylene glycol and glycerol buffer.

### 2.2. Antibody generation and selection

The mouse rapid immunization approach was used to generate mouse hybridomas secreting IgG monoclonal antibodies specific for adeno-associated virus 2 (AAV2).

Immunizations were performed with whole virus on female BALB/c mice with a total of 2*10^11^ recombinant AAV2-GFP per mouse over the immunization period for cell fusion and hybridoma cell line generation. Lymphocytes collected from immunized mice were harvested and counted and fused with murine SP2/0 myeloma cells in the presence of poly-ethylene glycol. The fusion product of up to 10^8^ lymphocytes was cultured using a single step cloning method.

Indirect ELISAs were used to screen for hybridomas that bind to AAV2. Primary screening was performed by indirect ELISA on whole live AAV2-GFP probing with secondary antibody for both IgG and IgM isotypes.

Monoclonal antibody binding affinity to AAV2 was assessed by calculating the ratio of virus/trapping Elisa with three fold titrations. Selected clones were then subcloned by plating parental clones into single cell colonies and antibody purified from culture supernatants using protein G columns. Purified antibody was stored in a carrier-free neutral low endotoxin buffer containing no azide.

### 2.3. Antibody conjugation to lipid microbubble vesicles

Antibodies were dialyzed into MES (2-ethanesulfonic acid) buffer, followed by the addition of 1.1 mg sulfo-NHS (N-hydroxysulfosuccinimide) (final concentration 5 mM) and 0.4 mg EDC (final concentration 2 mM). The mixture was allowed to react for 15 min, after which it was purified with an Amicon 10 kDa cutoff using MES buffer. The solution volume was reduced to 100–200 µl.

Associating antibodies to lipid-shelled microbubbles by covalent linkage is suitable for *in vivo* studies as they are stable in the bloodstream. Carboxy reactive antibodies were reacted with amine-PEG functionalized lipid vesicles for 1 h. The antibodies’ carboxy groups were covalently attached directly to the amine groups incorporated in the lipid vesicles and then purified of unbound antibody using dialysis with a 300 kDa cutoff over 48 h.

**Figure 1.**
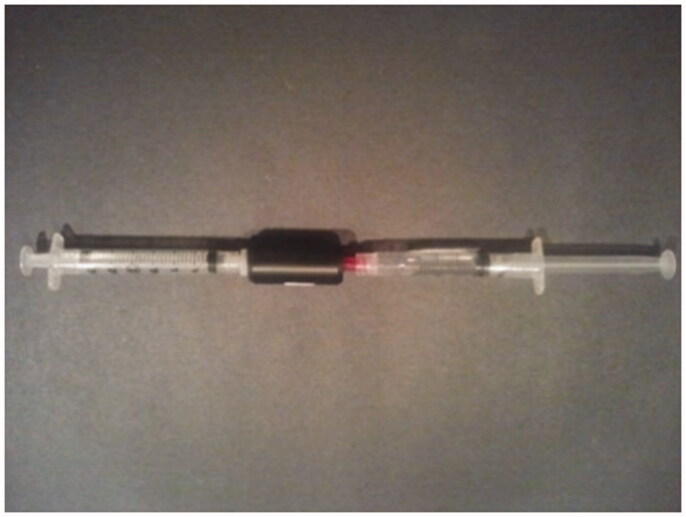
Artenga microbubble cartridges.

**Figure 2.**
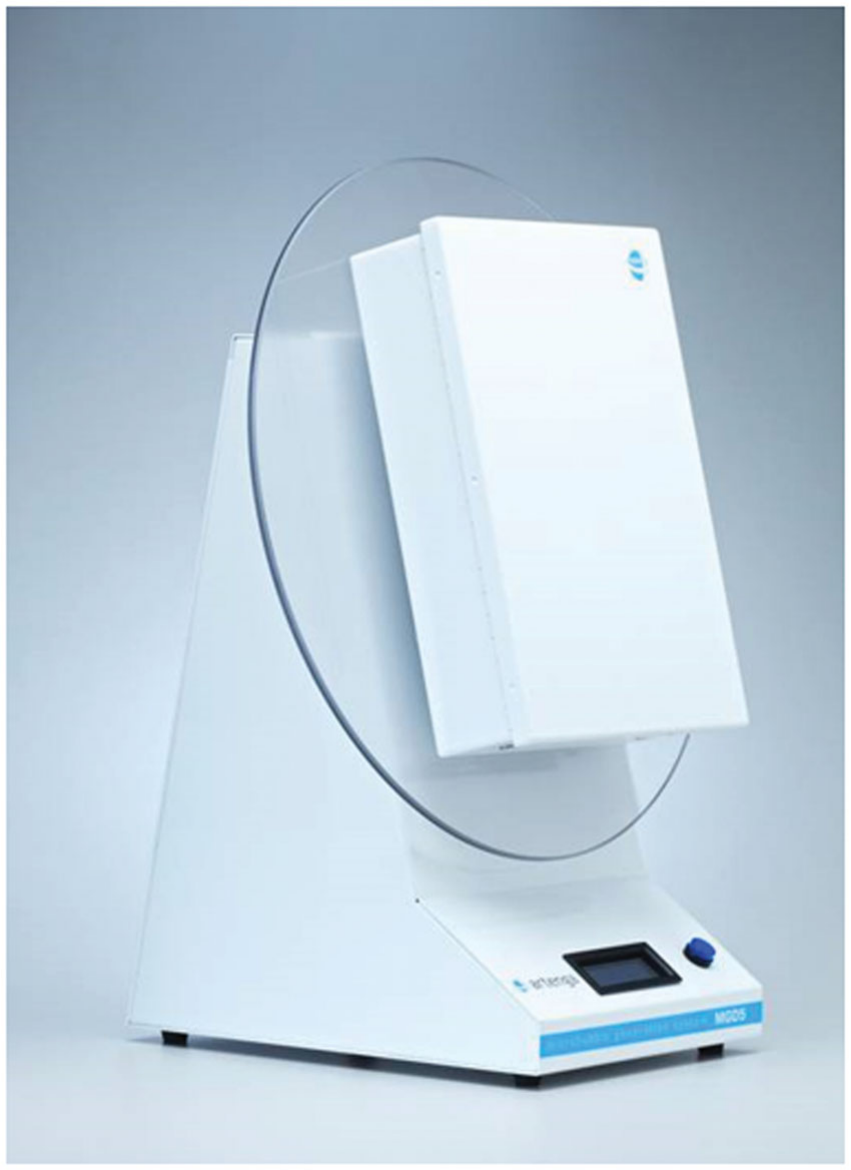
Artenga MGD5 microbubble generator.

The lipid-antibody solution and octofluoropropane (PFC) gas was then loaded into Artenga microbubble cartridges (see Figure 1) comprised of syringes and a micro-fluidic flow chamber. Cartridges were loaded into an Artenga MGD5 microbubble generator device (Figure 2) to induce reciprocating fluid and gas flow generation of microbubbles conjugated with 4C10 antibodies specific to AAV2. (Reference US8257338).

**Figure 3.**
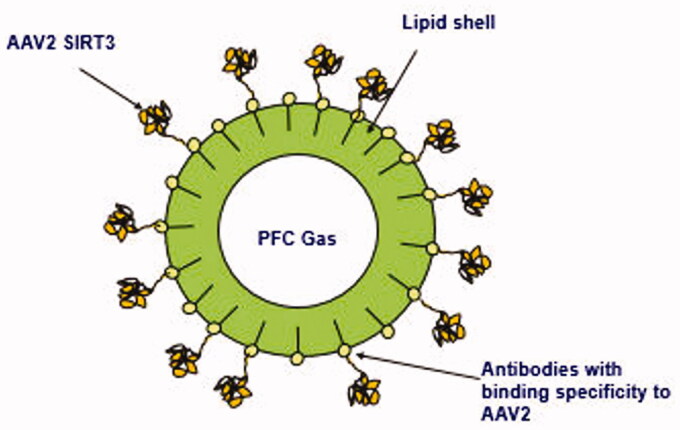
AAV2-SIRT3 Microbubble drug conjugate schematic.

### 2.4. Development of AAV gene therapy microbubble drug conjugates

AAV2-SIRT3 microbubble drug conjugates (MDCs) were synthesized as follows: lipid vesicles were formulated in propylene glycol buffer and monoclonal antibodies with binding specificity to AAV2 were produced. The antibodies were covalently conjugated to the lipid vesicles and perfluorocarbon gas filled, lipid shelled microbubbles generated. Next, AAV2-SIRT3 was added to permit binding of the AAV2-SIRT3 to the antibodies on microbubbles’ lipid shells (Figure 3). The solution was centrifuged and then reconstituted in clean buffer to remove AAV2-SIRT3 not bound to MDCs.

### 2.5. *In vitro* demonstration of efficacy of ultrasound + MBs-AAV2/eGFP to transduce SH-SY5Y cells

Localized destruction of microbubbles using ultrasound exposures within biological safety limits was done to dissociate AAVs from the microbubbles in the targeted area while maintaining the transduction capacity of the AAVs. Preliminary testing to demonstrate feasibility of this method used AAVs which transduce cells with Green Fluorescent Protein (GFP).

Microbubble cartridges containing the lipid-4C10 antibody was activated in the MGD5 microbubble generator (Artenga Inc, Ottawa, ON, Canada), which generated microbubbles conjugated with the 4C10 antibodies with binding specificity to AAV2. The microbubble solution was then centrifuged (8 min at 50 Gs), isolated and resuspended in a total of 1 mL of 1× phosphate buffered saline (PBS). This process was repeated three times. On the third time the microbubble solution was resuspended in a total volume of 0.9 mL of 1× PBS. The purpose of these washes was to remove any excess lipid-4C10 antibody that has not been incorporated into MBs. A volume of 100 µL of rAAV2/Tre-eGFP (6.7 × 10^12^ virus molecules/mL) was then added to the 0.9 mL of MBs solution and incubated for 15 min whilst gently mixing. After the incubation period the microbubble/AAV solution was centrifuged (8 min at 50 Gs), isolated and resuspended in a total of 1 mL of 1× PBS. This process was repeated three times to remove any unbound AAV, leaving only MBs conjugated with AAV (MBs-AAV2/eGFP).

**Figure 4.**
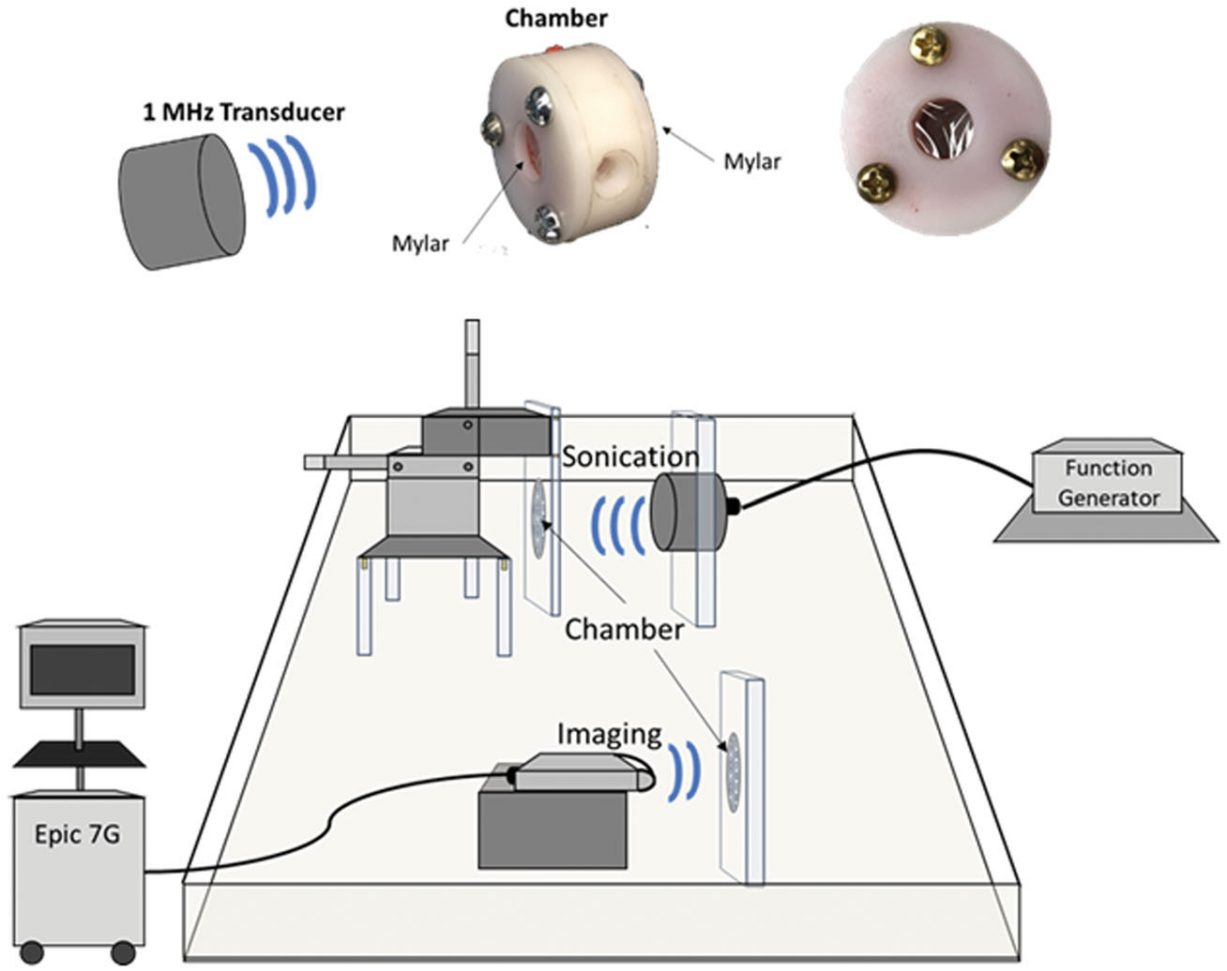
*In vitro* experimental setup for exposing MBs-AAV2 to ultrasound. The setup consists of a tank filled with degassed water, a 1 MHz spherically focused ultrasound transducer, a three-axis positioning system to hold the chambers, and an EPIQ 7G ultrasound imaging system with an L12-5 probe. The chambers are constructed out of Delrin and hold 1 mL of MBs-AAV2 solution between two mylar faces. There is one port for injection and extraction of the microbubble solution.

**Figure 5.**
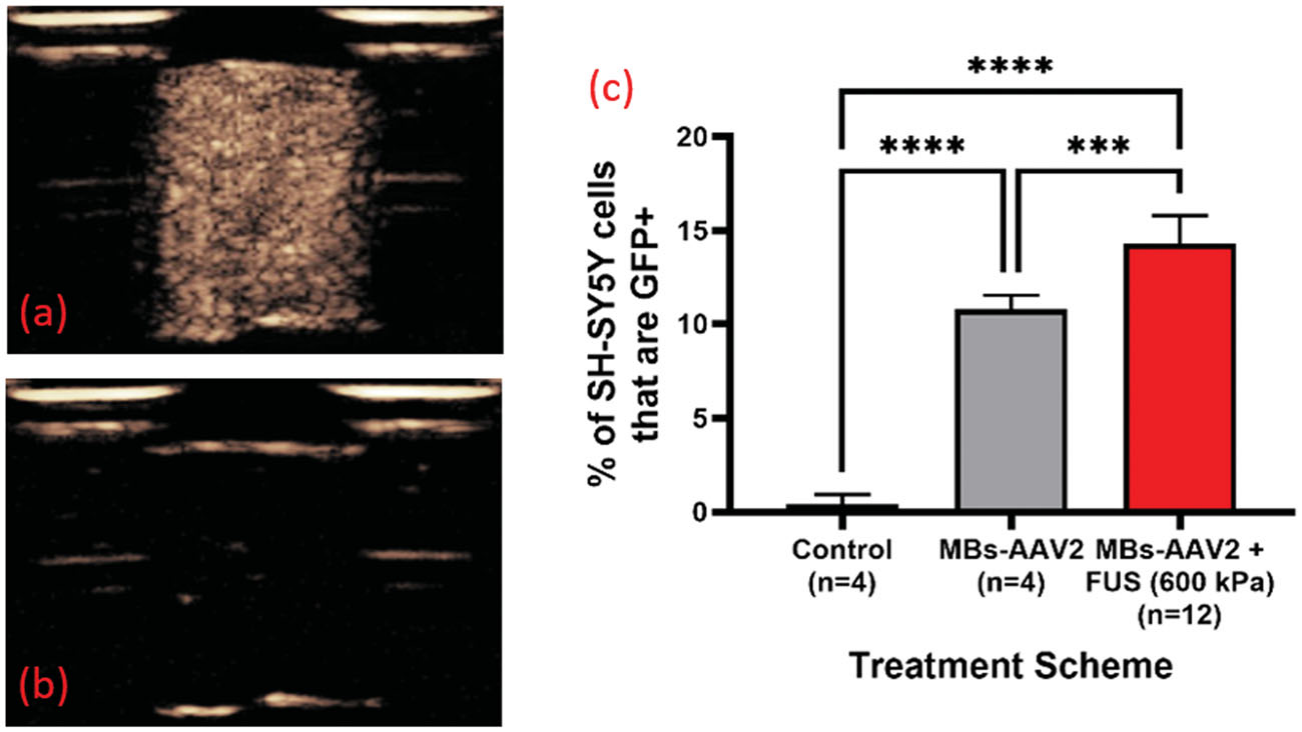
AAV2/eGFP conjugated MBs with FUS results in enhanced transduction of SH-SY5Y cells with eGFP. Contrast enhanced images of MBs-AAV2/eGFP (a) before FUS exposure and (b) post FUS exposure (600 kPa) demonstrating bubble destruction due to lack of contrast. (c) Destruction of MBs-AAV2/eGFP still retains ability to transduce SH-SY5Y cells. It should be noted that there was a significant increase in the transduction of cells when MBs-AAV2/eGFP were destroyed with FUS versus MBs-AAV2/eGFP that was not treated with FUS. Mean and standard deviations of the mean are plotted. ***p < 0.0005. ****p < 0.0001. MBs: microbubbles; AAV: Adeno-associated virus; FUS: focused ultrasound.

To assess whether MBs-AAV2/eGFP can transduce cells when sonicated, an experimental set-up was used consisting of the following apparatuses: a tank filled with degassed water, a 1 MHz spherically focused transducer (3.75-cm diameter, 15 cm focal length, 1.0 cm, −6 dB beam width at focus) (Valpy Fisher, Hopkinton, MA, USA), a three-axis positioning system to hold the chambers, and an EPIQ 7 G (Phillips, Amsterdam, Netherlands) ultrasound imaging system with an L12-5 probe (Figure 4). The chambers were constructed out of Derlin and hold 1 mL of solution between two mylar faces with one port for injection and extraction of the MBs-AAV2/eGFP solution. The chambers were autoclaved to maintain sterility due to cell culture requirements. To carry out sonication, 2 µL of MBs-AAV2/eGFP conjugate was added to 1998 µL of cell culture medium consisting of a 1:1 EMEM medium (Wisent, Catalog No. 30-2003) and HAM’s F12 medium (Wisent, Catalog No. 318-010-CL), supplemented with 10% fetal bovine serum (Wisent, Catalog No. 080-450). The chamber was then filled with 1 mL of the newly diluted MBs-AAV2/eGFP in cell culture medium and was imaged with the EPIQ 7G ultrasound machine in contrast mode and then subjected to ultrasound treatment. The sonication scheme employed was as follows: 10 ms long pulses at 600 kPa, repeated every second for a duration of 1 min. The chamber was then removed and gently mixed and subjected once more to the sonication scheme. After two rounds of sonication were completed, the chamber was imaged under contrast mode with the EPIQ 7G ultrasound machine to confirm the destruction of the MBs. In addition to sonicating the MBs-AAV2/eGFP within the chamber, two other treatment groups were evaluated. The first group was control, which consisted of cell culture media within the chamber being exposed to FUS (at 600 kPa). The second group was MBs-AAV2/eGFP within the chamber but not being exposed to FUS (Figure 5).

After treatment the MBs solution was removed from the chamber and placed into a 3 mL syringe and centrifuged for 8 min at 50 Gs and the supernatant was isolated from the ‘froth’ (any remaining undestroyed MBs). Approximately 190 µL of supernatant was incubated with 20,000 SH-SY5Y cells (ATCC, Catalogue No. CRL-2266) in a total volume of 200 µL for 72 h in a 96 well plate. After the incubation period, the cells were trypsinized and the nucleic acid-binding dye DAPI (BD Biosciences, Catalog No. 564907) was added to exclude dead cells from live cells. Flow cytometry on the SH-SY5Y cells was performed using an LSR II (BD Biosciences) and analyzed offline on Flowjo Software version 10.00 (Tree Star Inc., Ashland, OR, USA). The total GFP + SH-SY5Y cells was identified as a percentage of the total live cell population.

### 2.6. Conjugation of AAV2.CB7.CI.Sirt3-myc.WPrE.rBG to activated microbubbles

Microbubble cartridges containing microbubbles, 4C10 antibody linker, and activator (Artenga Inc., Canada) were incubated for 12 min to activate the microbubble cartridges. The microbubbles were then decanted to remove large unwanted microbubbles, and centrifuged for 8 min at 55 g. The resulting supernatant was discarded to remove free lipids and unbound antibody linker, and the activated microbubbles were resuspended in a total volume of 0.9 mL of sterile PBS. One hundred microliter of AAV2.CB7.CI.Sirt3-myc.WPRE.rBG (UPenn Vector Core, USA) at a concentration of 9.86 × 10^12^ GC/mL was added and incubated with the activated microbubbles for 15 min, followed by an 8 min centrifugation at 55 g. The supernatant containing unbound AAV2 was discarded, and the conjugated microbubbles were resuspended in a total volume of 1 mL of sterile PBS.

### 2.7. *In vivo* MR-guided-focused ultrasound overexpression of AAV2.SIRT3-myc

Male Sprague-Dawley rats weighing 350–400 g (Charles River, Canada) were anesthetized with 2–3% isoflurane and tail-vein catheterized with a 22G catheter. The rats were administered 0.03–0.04 of gadolinium contrast agent Gadovist^®^ (Schering AG, Berlin, Germany) at a dose of 0.2 mL/kg and were and secured in a supine position on a sled that is compatible and spatially co-registered with both the MR (7 T MRI BioSpec 70/30 USR, Bruker, Billercia, MA, USA) and the ultrasound treatment system (LP100, FUS instruments Inc., Toronto, ON, Canada).

The top of the rat’s skull was coupled with a layer of ultrasound gel to an acoustically transparent polyimide water pack that had its base submerged in a tank of degassed deionized water, which held the therapeutic transducer. The rats were subjected to MR imaging in which T2-weighted MR images were obtained and the spatial coordinates for the striatum (2 adjacent treatment locations) and substantia nigra (1 treatment location) were chosen in the animal’s right hemisphere.

FUS treatments on the striatum and substantia nigra were performed with the LP100 system using a spherically focused, 580 kHz, F0.50NO.8D75CH25-1 transducer (0.8 focal number; 7.5 cm diameter) with a −6 dB beam width and 16 mm wide PZT hydrophone.

Microbubble solution containing 4C10 antibody was generated and then centrifuged (8 min at 50Gs), isolated and resuspended in a total of 0.9 mL of 1× PBS. The purpose of this was to remove any excess lipid-4C10 antibody that has not been incorporated into MBs. A volume of 100 µL of rAAV2/CB7.CI.Sirt3-myc.WPRE.rBG (AAV2/Sirt3 for short) (9.86 × 10^12^ virus molecules/mL) was then added to the 0.9 mL of MBs solution and incubated for 15 min whilst gently mixing. After the incubation period the microbubble/AAV solution was centrifuged (8 min at 50Gs), isolated and resuspended in a total of 1 mL of 1× PBS. This process was done to ensure that we were left only with MBs conjugated with AAV (MBs-AAV2/Sirt3).

Immediately prior to FUS treatment, 0.3–0.4 mL of MBs-AAV2/Sirt3 was injected followed by a 0.5 mL saline flush. FUS treatment entailed 10 ms bursts at 0.4 MPa and pulse repetition frequency of 1 Hz, every 1 s for a total of 120 s.

Post-FUS treatment, 30 µL of a 1:10 dilution of the gadolinium-based contrast agent was injected followed by a 0.5 mL saline flush. Following contrast agent administration, T1-weighted images were then obtained to confirm unilateral BBB opening in the striatum and substantia nigra. Animals were then allowed to recover in their cage with gentle stimulation.

### 2.8. Brain tissue harvest and cryosectioning

Two weeks following the FUS procedure, animals were humanely euthanized using isoflurane and cardiac perfusion with ice-cold PBS. Brain tissue was fixed in 4% PFA in PBS for 24 h, washed with ice-cold PBS for 10 min three times, and soaked in 30% sucrose in PBS for 2–3 days at 4 °C until the brains sink in the solution. Coronal sections 40 μm in thickness were cryosectioned and stored in cryoprotection solution at −20 °C for analysis at a later date.

### 2.9. Immunofluorescence labelling and imaging

Cryoprotection solution was washed off of the tissue sections using three 10 min washes in PBS-T (0.1% Triton X). Sections were blocked for 1 h at room temperature, shaking in 5% normal goat serum (NGS) in PBS-T. The block was then removed, and the sections were incubated overnight at 4 °C, shaking in mouse anti-myc (1:2000, Cell Signaling Technologies 2276S) and rabbit anti-Sirt3 (1:200, Cell Signaling Technologies 2627S) primary antibodies diluted in 1% NGS in PBS-T. Following the overnight incubation, the sections were washed three times for 5 min in PBS-T, and then incubated for 2 h at room temperature, shaking and protected from light in Alexa Fluor 488 goat anti-mouse (1:500, Jackson Immuno 111-585-144) and Alexa Fluor 594 goat anti-rabbit (1:500, Jackson Immuno 115-545-003) secondary antibodies diluted in 2% NGS in PBS-T. Sections were then washed in PBS three times for 5 min, and incubated for 10 min in DAPI before washing three additional times for 5 min in PBS. Sections were mounted on microscope slides (VWR CA48311-703), allowed to anneal, and rinsed with double distilled water for 2 min. Lastly, sections were coverslipped with fluorescent mounting media (Agilent S3023) and glass coverslips (VWR CA48393-081), and sealed with clear enamel. Microscope slides were stored at 4 °C protected from light until imaged using a WaveFX Spinning Disk Confocal microscope (Quorum Technologies, Canada).

## 3. Results and discussion

### 3.1. *In vitro* demonstration of efficacy of ultrasound + MBs-AAV2/eGFP to transduce SH-SY5Y cells

Interpretation for Results/Discussion: In the treatment group with MBs-AAV2 with no FUS there is a significant proportion of transduction of SH-SY5Y cells despite MBs-AAV2 being removed from solution (via centrifugation) prior to incubation with SH-SY5Y cells. A potential reason for this is that there might be dissolution of the MBs, leading to free AAV2 to transfect SH-SY5Y cells. This can happen at two stages, the first being the brief period (∼5 min) that MBs-AAV2 spends in the chamber before being centrifuged and the second is in the centrifugation process to remove any undestroyed MBs after sham treatment in the chamber. However, it should be noted that MBs-AAV2 that were treated with FUS resulted in a significantly higher percentage of SH-SY5Y cells that are GFP + compared to MBs-AAV2 with no FUS. This suggests that FUS is able to free more AAV2 from MBs-AAV2 complexes to transfect SH-SY5Y cells. This provides evidence to localized delivery of AAV with FUS when targeting nuclei of the brain.

### 3.2. *In vivo* MR-Guided-Focused ultrasound overexpression of AAV2.SIRT3-myc

**Figure 6.**
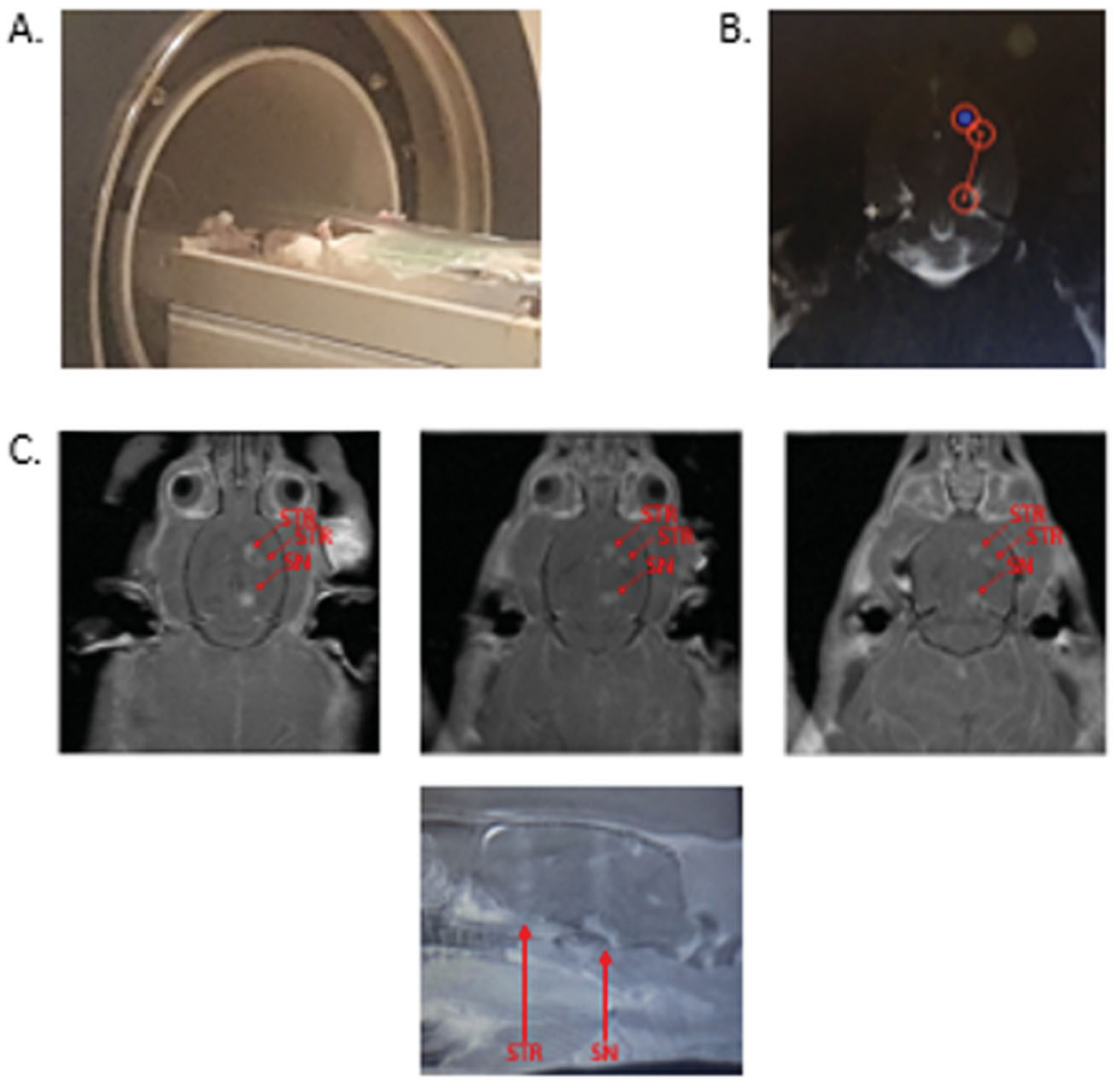
MR-guided FUS induced permeabilization of the blood brain barrier. (A) Anesthetized rat in the MRI with rodent attachment. (B). T1-weighted image following gadolinium contrast agent (0.2 mL/kg, GE Healthcare) administration to locate and target the striatum (two sites) and substantia nigra (one site). (C). T1-weighted images following tail-vein infusion via catheter of AAV2-SIRT3-myc conjugated to microbubbles (300–400 µl) and application of sonication at targeted sites in the striatum (ST) and substantia nigra (SN) (0.4 MPa fixed pressure, 1 ms pulse length, 1 Hz pulse repetition frequency, 120 s duration). MR imaging with gadolinium shows FUS-targeted areas as bright regions (red arrows), indicating BBB permeabilization, and that gadolinium diffused into the SNc and ST. *n* = 5.

**Figure 7.**
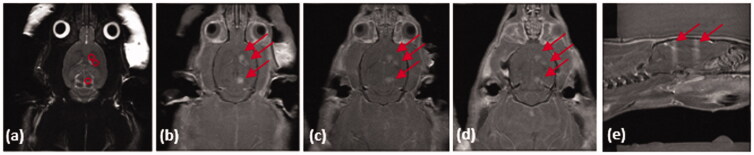
MRIgFUS with AAV2-Sirt3 conjugated MBs causes temporary opening of the BBB in striatum and substantia nigra. (a) The striatum (2 treatment points) and the substantia nigra (1 treatment point) were targeted unilaterally from T2-weighted images (targets are outlined by red circles). Following FUS treatment (US parameters described in material and methods section), the contrast agent gadolinium was given intravenously and (b–d) T1W transverse and (e) saggital images were taken in which the red arrows demonstrate BBB opening. It should be noted that only the sonicated hemishphere demonstrates BBB opening.

To determine whether FUS delivered AAV2.SIRT3-myc – conjugated microbubbles to brain regions affected in Parkinson’s disease, male Sprague Dawley rats were anesthetized and placed on a rodent MRgFUS positioning system (Figure 6(a)). MRgFUS was used to target the striatum (2 sonications) and SNc (1 sonication; Figure 6(b)). BBB permeability within the treated area was assessed by MRI, using the gadolinium-based contrast agent into the brain (Figure 6c). When targeting both the striatum and the SNc, an influx of gadolinium was visualized, after treatment, demonstrating the BBB was permeabilized within a 2 mm diameter viewed in a horizontal plane. Permeabilization of the BBB was confirmed in coronal and sagittal views (Figure 6c and Figure 7(a-e)).

**Figure 8.**
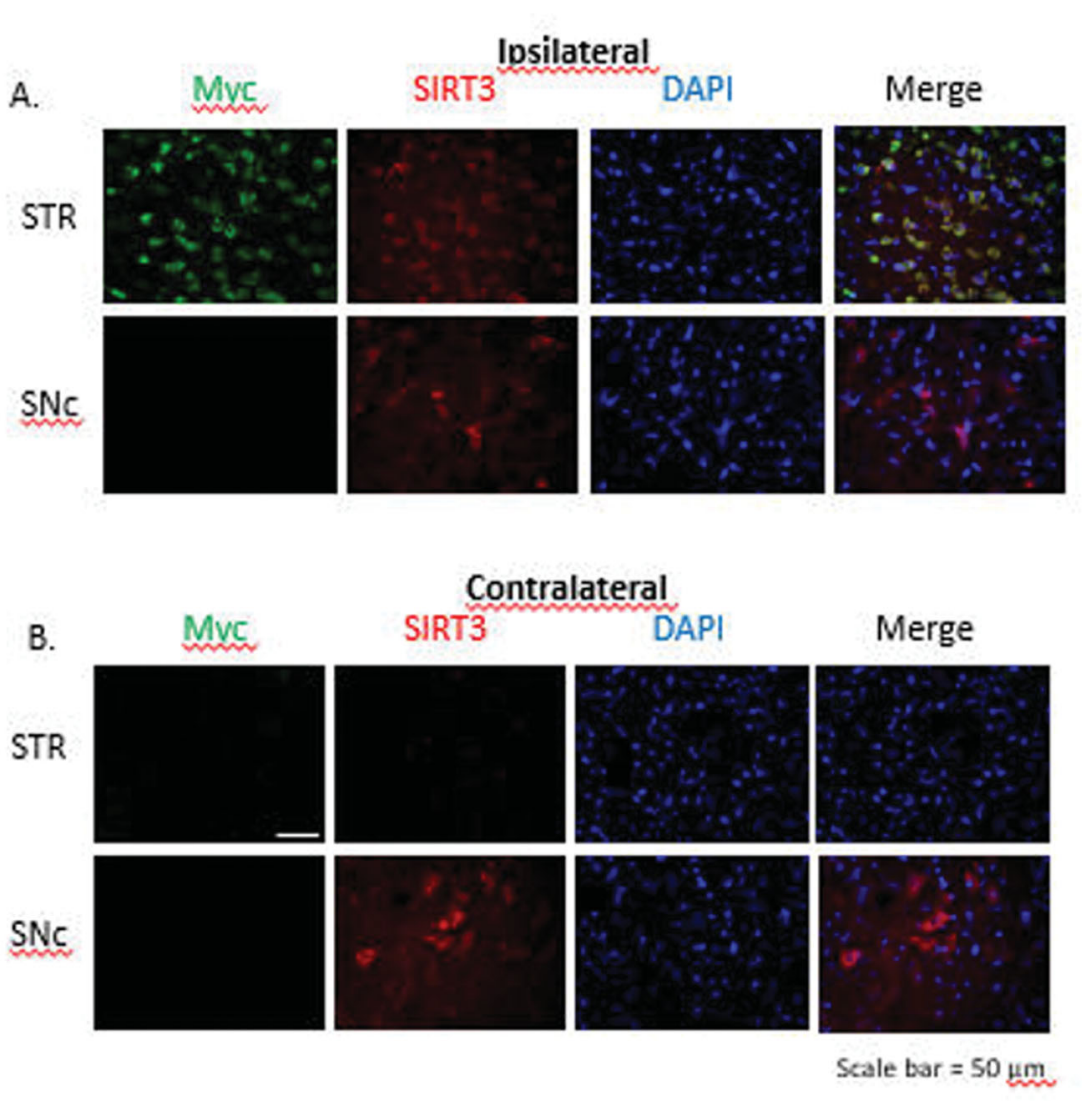
Immunofluorescent images to show transduction of SIRT3-myc following MRg-FUS induced BBBB permeabilization. Fourteen days following MR-g-FUS mediated BBB permeabilization and administration of AAV.SIRT3-myc, animals were euthanized and brains removed, then processed for immunofluorescent analysis. In the hemisphere ipsilateral to FUS application, SIRT3-myc was successfully expressed in the striatum, but not the SNc. No SIRT3-myc expression was observed in the contralateral hemisphere. Antibodies: Myc (SIRT3-myc), SIRT3 (endogenous and ectopic SIRT3), and DAPI (nucleus). *n* = 5.

To determine the efficiency of SIRT3-myc transduction following MR-g-FUS mediated delivery of AAV2.SIRT3-myc, animals were euthanized, brains removed, and immunofluorescence performed. MR-g-FUS mediated BBB permeabilization resulted in SIRT3-myc transduction in the striatum, but not the SNc ipsilateral to the sonication site. Qualitative studies showed elevated levels of SIRT3-myc compared to endogenous SIRT3, as determined using antibodies against myc (green) and SIRT3 (red) respectively (Figure 8).

These studies show that MR-g-FUS mediated permeabilization of the BBB is a useful method for the delivery of AAV2.SIRT3-myc. In addition, conjugated microbubbles are effective as a delivery method for AAVs. Currently it is unknown why there was no efficient biodistribution of SIRT3-myc in the SNc, while there was in the striatum, when gadolinium showed sufficient BBB permeabilization. Future studies will involve optimization of FUS parameters to hopefully result in SIRT3-myc transduction in the SNc.

## 4. Conclusions

These results indicate the feasibility of developing clinically scalable, microbubble drug conjugates employing AAV2-SIRT3 and other viral vector gene therapies to treat neurodegenerative disease. The MDCs are infused intravenously, focused ultrasound targets diseased regions of the brain to noninvasively permeabilize the blood–brain barrier and to release the viral gene therapy for transduction.
